# Norms for Clinical Use of CXM, a Real-Time Marker of Height Velocity

**DOI:** 10.1210/clinem/dgaa721

**Published:** 2020-10-09

**Authors:** Ryan F Coghlan, Robert C Olney, Bruce A Boston, Daniel T Coleman, Brian Johnstone, William A Horton

**Affiliations:** 1 Research Center, Shriners Hospitals for Children, Portland, Oregon; 2 Division of Endocrinology, Nemours Children’s Specialty Care, Jacksonville, Florida; 3 Department of Pediatrics, Oregon Health & Science University, Portland, Oregon; 4 Graduate School of Social Service, Fordham University, New York, New York; 5 Department of Orthopaedics & Rehabilitation, Oregon Health & Science University, Portland, Oregon; 6 Department of Molecular & Medical Genetics, Oregon Health & Science University, Portland, Oregon

**Keywords:** CXM, type X collagen, biomarker, height velocity, growth, bone growth

## Abstract

**Context:**

Height velocity (HV) is difficult to assess because growth is very slow. The current practice of calculating it from measurements taken at several-month intervals is insufficient for managing children with growth disorders. We identified a bone growth by-product (collagen X biomarker, CXM) in blood that in preliminary analysis in healthy children correlated strongly with conventionally determined HV and displayed a pattern resembling published norms for HV vs age.

**Objective:**

The goal was to confirm our initial observations supporting the utility of CXM as an HV biomarker in a larger number of individuals and establish working reference ranges for future studies.

**Design, Settings, and Participants:**

CXM was assessed in archived blood samples from 302 healthy children and 10 healthy adults yielding 961 CXM measurements. A total of 432 measurements were plotted by age, and sex-specific reference ranges were calculated. Serial values from 116 participants were plotted against observed HV. Matched plasma, serum, and dried blood spot readings were compared.

**Results:**

A correlation of blood CXM with conventional HV was confirmed. Scatter plots of CXM vs age showed a similar pattern to current HV norms, and CXM levels demarcated the pubertal growth spurt both in girls and boys. CXM levels differed little in matched serum, plasma, and dried blood spot samples.

**Conclusions:**

Blood CXM offers a potential means to estimate HV in real time. Our results establish sex-specific, working reference ranges for assessing skeletal growth, especially over time. CXM stability in stored samples makes it well suited for retrospective studies.

We have recently identified a fragment of type X collagen as a potential real-time biomarker for height velocity (HV) in growing children (1). Type X collagen is normally produced by hypertrophic chondrocytes during endochondral ossification, which in growing children occurs almost exclusively in active growth plates (2-4). The biomarker is a degradation by-product of this process and released into the circulation in proportion to overall growth plate activity.

Our original publication described the identification and detailed characterization of the biomarker CXM (collagen X biomarker) and the development of a sensitive immunoassay to measure CXM in blood (1). Based on its growth plate origin, we posited that CXM concentrations plotted against age would display a pattern similar to established HV curves (5-7). Preliminary analysis of data from 83 normally growing children plotted in this fashion showed this pattern. Similarly, a strong correlation was observed when CXM levels were plotted against HV calculated from conventional 6-month stadiometer readings from 44 individuals.

Although these data strongly support the concept that growth velocity can be estimated in real-time using CXM, the number of measurements from which the curves were constructed was limited, especially for children of pubertal age. To address this concern, we analyzed CXM from a total of 302 normally growing, healthy individuals with no known risk factors for impaired growth to generate data used for cross-sectional analysis and growth velocity correlations. Moreover, we optimized the originally reported CXM assay and retested our 44 previously analyzed samples using the optimized assay. Data generated from testing these individuals increased the total CXM values from healthy, normally growing individuals analyzed to 432 for cross-sectional analysis and 116 for growth velocity correlations.

Compared with our initial report, the scatterplots for CXM determined by the optimized assay vs age showed the same characteristics and comparable CXM measurements as previously reported. The samples in this study were run on an optimized CXM assay that used prediluted calibrator and quality control sets to limit calibrator and ultimately sample variation over time. In this expanded study there were substantially more data points in the pubertal and prepubertal age ranges, allowing for a clear demarcation in the timing of the pubertal growth spurt both in girls and boys. Similarly, the correlation of CXM values to HV calculated from conventional 6-month incremental growth determinations remained strong. Our new results support the conclusion that CXM is a real-time biomarker for growth velocity, and they provide working reference ranges for future investigations using CXM to assess skeletal growth.

## Materials and Methods

### Study design

The goals of this investigation were to validate CXM as a marker for HV at time of measurement, to describe modifications that optimize the CXM assay, and to establish reference ranges for CXM values in healthy, normally growing infants and children with no known risk factors for impaired growth. The optimized CXM assay was used to reanalyze samples from Shriners Hospitals for Children (SHC) and Oregon Health & Science University (OHSU) in Portland, Oregon, used for the previous CXM study (1), as well as additional samples from Nemours Children’s Specialty Care, Jacksonville, Florida.

### Study procedures

Acquisition of serum, plasma, and dried blood spot (DBS) samples collected from SHC and OHSU clinics in Portland, Oregon, including institutional review board approval was described previously (1). The collection of serum and plasma samples from Nemours Children’s Specialty Care, Jacksonville, Florida, was described by Olney et al (8). The study was approved by the Nemours Florida Institutional Review Board, including the subsequent research use of the samples.

Forty-two individuals were recruited from SHC, 220 from Nemours Children’s Specialty Care, and 40 from OHSU. Of those enrolled, 190 participants had single appointments when plasma, serum, or DBS and biometric measurements were collected. Ninety-four participants were evaluated twice approximately 6 months apart when plasma and serum samples were collected. Eighteen individuals were sampled up to 3 times at time points of 0, 6, and 12 months. Two male samples yielded HV of greater than 20 cm per year. They were excluded from HV analysis as outliers because their extreme rate of growth was assumed to be due to measurement error. The characteristics of the participants involved in the study are summarized in Table 1; detailed information is provided in Supplemental Table 1 (9).

**Table 1.** Summary of participants

Site	Nemours	SHC	OHSU	Total
Total participants				
No.	243	42	40	302
Sex, M:F	123:97	25:17	15:25	163:139
Age, y^*a*^	1.9 to 19.8	0.1 to 13.8	0.2 to 15.4	0.1 to 19.8
Height range (*z* score)^*a*^	–1.8 to 3.4	–3.5 to 2.0	–3.5 to 3.0	–3.5 to 3.4
Participants’ HV calculated				
No.	88	–	22	110
Sex, M:F	50:38	–	6:16	56:54
Age, y^*a*^	3.0 to 19.8	–	1.3 to 15.0	1.3 to 19.8
Height range (*z* score)^*a*^	–1.5 to 2.6	–	–1.2 to 2.7	–1.5 to 2.7

Race/Ethnicity was recorded only at the Nemours site: White:Black:Hispanic:Asian:Other:Unknown 154:48:13:1:2:2.

Abbreviations: F, female; HV, height velocity; M, male; Nemours, Nemours Children’s Specialty Care; OHSU, Oregon Health & Science University; SHC, Shriners Hospital for Children.

^*a*^At baseline.

Blood samples were collected from 10 nongrowing adults from the SHC site for control purposes. Additionally, blood samples from 34 individuals followed in growth clinics were used for the plasma-serum-DBS comparisons only.

Heights were measured on standing, wall-mounted stadiometers (Perspective Enterprises at SHC and Holtain Ltd at Nemours) and calibrated daily by a standard 100-cm rod. At both centers, measurements were completed in a clinical setting in the Pediatric Endocrine and Diabetes clinics by medical assistants specifically trained in accurate measurement techniques. Height and weight measurements were recorded at the time of sampling for each patient. For participants with serial height measurements at least 6 months apart, annualized HV was calculated using the change in height measurements.

As described in the original publications, the blood sampling protocols differed at the sites (1, 8). Samples were taken at the beginning of the study interval for the Nemours samples and at the end or the interval for the SHC and OHSU samples. Almost all samples were taken in the morning for the Nemours participants; many were collected in the afternoon for the SHC and OHSU participants.

Sample sizes for tests of CXM marker to HV associations were not determined by a priori power analyses because this was an observational study using convenience samples.

Plasma and serum samples were processed in vacutainers (Beckton-Dickson No. 368036 and No. 367983, respectively), aliquoted into microcentrifuge tubes, and stored immediately at –20°C or –80°C.

### Analytical procedures

The original CXM assay protocol was described previously (1). It is abbreviated here except for modifications relevant to its optimization.

### Serum, plasma dilution, and assay procedure

All serum and plasma samples were thawed and diluted in sample diluent at 1:200 for individuals younger than 18 years or 1:20 for participants older than 18 years. All diluted samples were assayed in duplicate using the same batch lot of plates, calibrators, and quality controls (QC). The calibrator consisted of recombinant noncollagenous domain 1 of human type X collagen purchased from BioMatik.

### Dried blood spot sampling

DBS samples were obtained by finger sticks and spotting onto Whatman 903 Protein Saver Cards. DBS cards were then dried for 1 to 4 hours at room temperature, placed in resealable bags containing desiccant packets, and stored at –20°C until assayed. All samples included in this study were assayed in a blinded fashion in duplicate. Information pertaining to these samples can be found in Supplemental Table 1 (9). All data were graphed using GraphPad Prism software, version 7.03.

### Dried blood spot elution and assay procedure

**Figure 1.**
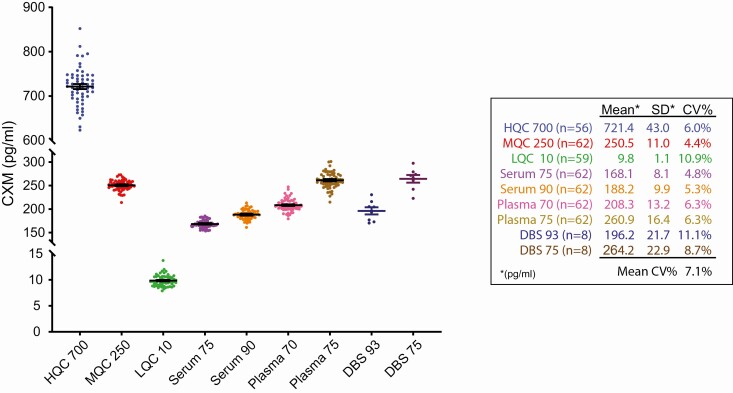
Quality control data plotted by standard. Spiked rNC1, plasma, and serum control data over 31 separate collagen X biomarker (CXM) enzyme-linked immunosorbent assay plates are shown. Mean ± 1 SE bars are overlaid on each data set. Data shown on right.

One 3.1-mm punch was taken per pediatric DBS spot and eluted with 250 µL of sample diluent in the well of a sealed polypropylene microplate. The plate was incubated overnight at 4°C on ice to reduce condensation. The elution plate was then placed on a shaker at 450 rpm for 10 minutes at room temperature. Each sample (100 µL) was then measured in duplicate and the CXM concentration determined from a serial diluted rNC1 calibrator curve using 4 Parameter Logistic nonlinear regression model fit from BioTek Gen5 software (*R*^2^ > 0.95 was acceptable). DBS quality controls created in the initial data collection were run with each DBS assay plate and data plotted in Fig. 1. Each result was multiplied by its associated dilution (calculated dilution factor assumes 1.67 µL plasma per spot assayed) for its equivalent nanogram per milliliter (ng/mL) concentration.

### Optimization of collagen X biomarker assay

rNC1 purchased from BioMatik was reconstituted, actual concentration determined using amino acid analysis and Qbit 2.0 fluorometer protein analyzers, then diluted to a stock concentration of 700 ng/mL for use in calibrator and quality control–spiked sample preparations. Prior to assay optimization, calibration curves for the CXM assay were prepared by serially diluting 800 pg/mL rNC1 calibrator into sample diluent immediately before running an assay. It was discovered that the rNC1 calibrator can be difficult to dilute with low levels of variance unless vigorously vortexed, therefore a prediluted set of calibrators was created for assay optimization. A total of 200 mL of each calibrator (800, 400, 200, 100, 50, 25, 12.5, and 0 pg/mL) was created by diluting the rNC1 stock in sample diluent from 700 ng/mL stock preparations. A total of 675 µL of each level was aliquoted into 1.1-mL strip tubes and stored at –20°C to use in future assays. Serum and plasma quality control samples were created by diluting freshly thawed serum or plasma 1:200, aliquoting, and storing in a similar fashion to the calibrators. Before performing a CXM assay, a set of each calibrator and controls was thawed at room temperature, vortexed vigorously for 1 minute, then centrifuged at 1000 rpm for 1 minute to remove droplets that may have adhered to the caps while vortexing. An 8-well multichannel pipette was then used to reverse-pipette 100 µL of each calibrator or quality control set into a CXM 96-well enzyme-linked immunosorbent assay (ELISA) plate in duplicate. A 675-µL set of strip tubes contains enough sample to run three 96-well CXM ELISA plates. This batch production both of calibrators and controls limited the amount of variance that occurs through serial dilution of one calibrator and repeat dilution of each serum or plasma control over time. This lot of calibrators and controls will be used to verify and validate calibrator and QC preparations in the future. The data from our previous publication relied on calibration curved from serially diluted stocks of rNC1 for each run, potentially increasing interassay variability.

Two types of QC samples were prepared for assessing the interassay and intra-assay variance as well as validating each CXM assay run. rNC1 controls were created by spiking rNC1 stock into sample diluent in a method similar to the calibrator preparation at concentration levels between the calibrators, namely 700 pg/mL (HQC 700), 250 pg/mL (MQC 250), and 10 pg/mL (LQC 10). Serum and plasma QCs were created by diluting human serum and plasma samples of children with sufficient quantity for bulk dilution 1:200 and aliquoting into strip tubes.

### Data analysis

CXM vs age data tables for girls and boys (Supplemental Table 1) were entered into the R software package loaded with the Generalized Additive Model for Location, Scale and Shape (GAMLSS) v5.1 to 4 statistical package (9, 10). Subsets of the data were analyzed for sex, and the cutoff for age was set at 21 years. Within the GAMLSS package, the LMS method using ST3 curve distribution calculated with lowest global deviance to generate centiles curves for the data from girls, whereas BCCGo was used for boys. Centile curves were plotted with normal data superimposed over curves in Prism software version 0.8.3.0 for Windows (GraphPad Software; www.graphpad.com).

Simple linear regression models correlations and the Kruskal-Wallis test for group differences with the Dunn test follow-up were computed in Prism version 0.8.3.0 and Stata 14 (StataCorp; www.stata.com). The difference in correlation coefficients was tested using Fisher *Z* transformation (11).

## Results

We performed a variety of technical validation tests after assay optimization to verify that the assay dynamics met accepted standards. CXM calibrators and controls as described in the assay optimization section were run on each plate and were used to generate data for this study. Fig. 1 shows the control data generated from 31 96-well ELISA plates run in this study. Average interassay coefficient of variation percentage for serum and plasma controls ranged from 4.8% to 6.3% with a similar variance for HQC 700 (700 pg/mL) and MQC 250 (250 pg/mL). The lowest QC level, LQC 10 (10 pg/mL) exhibited the highest variation of 10.9%, which was expected because this control is very close to the previously determined lower limit of quantification of 5.4 pg/mL. Overall, these data showed a low level of interassay and intra-assay variance both for spiked-rNC1 and diluted serum and plasma samples. The average intraassay variation for all serum, plasma, and DBS data was 3%, 4% and 4%, respectively.

**Figure 2.**
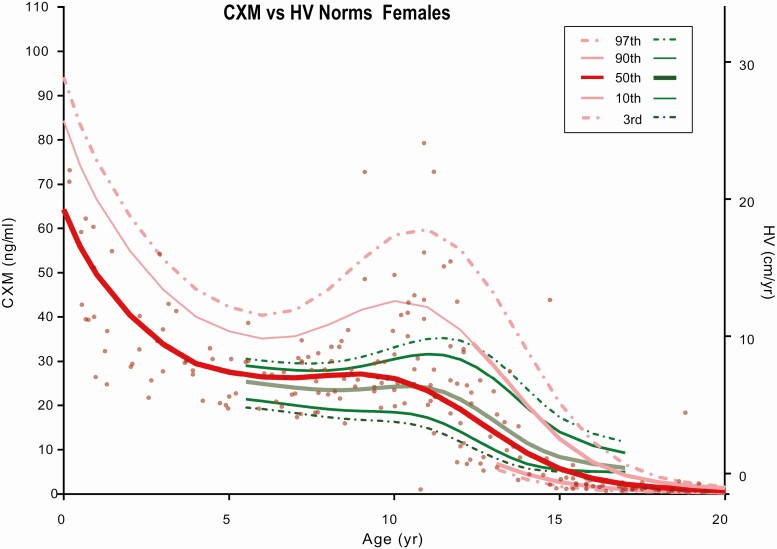
Collagen X biomarker (CXM) values by age for females. CXM percentile reference curves from the third to 97th percentiles for females generated using LMS analysis. Green curves are standard height velocity (HV) curves from the third to 97th percentiles taken from Kelly et al (7). HV vs CXM ng/mL equivalent levels were added as a right y-axis according to the data Fig. 4B from this manuscript.

**Figure 3.**
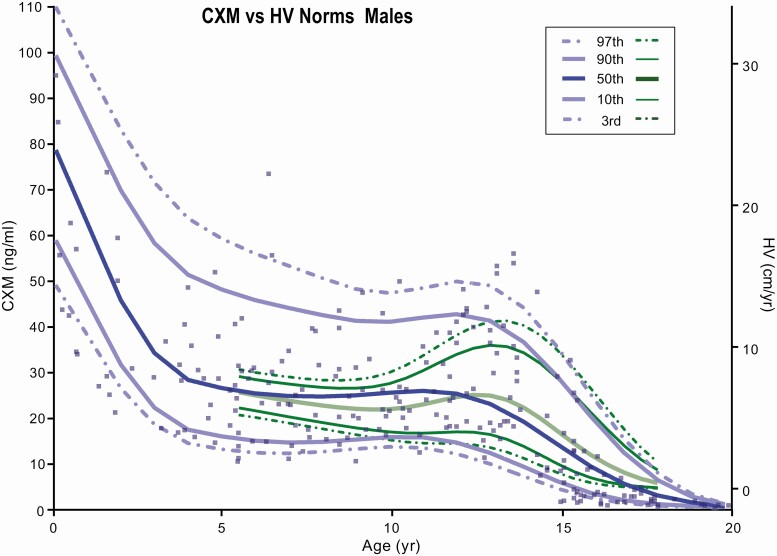
Collagen X biomarker (CXM) values by age for males. CXM percentile reference curves from the third to 97th percentiles for males generated using LMS analysis. Green curves are standard height velocity (HV) curves from the third to 97th percentiles taken from Kelly et al (7). HV vs CXM ng/mL equivalent levels were added as a right y-axis according to the data Fig. 4B from this manuscript.

The cross-sectional CXM data along with percentile curves (97%, 90%, 50%, 10%, 3%) for each sex were plotted in a scatterplot by age and CXM concentration in Figs. 2 and 3. Percentile curves were calculated using the LMS method within the R software package (12), which uses the mean (M) and coefficient of variation (S) to summarize the CXM vs age data into a smooth (L) curve (13). The well-established HV percentile curves published by Kelly and colleagues (7) were superimposed on CXM data in Figs. 2 and 3 in green. Similar to established norms, the pubertal growth spurt identified by CXM values was approximately 2 to 3 years earlier for girls compared with boys. The ages of peak growth velocity of our study participants were close to those previously reported for girls and boys by Kelly et al (7).

As shown in Figs. 2 and 3, CXM values decrease in a linear fashion during the transition from the peak HV through the postpubertal growth cessation to nongrowing adults. CXM levels were all less than 1 ng/mL in the 10 participants older than 20 years involved in this study.

**Figure 4.**
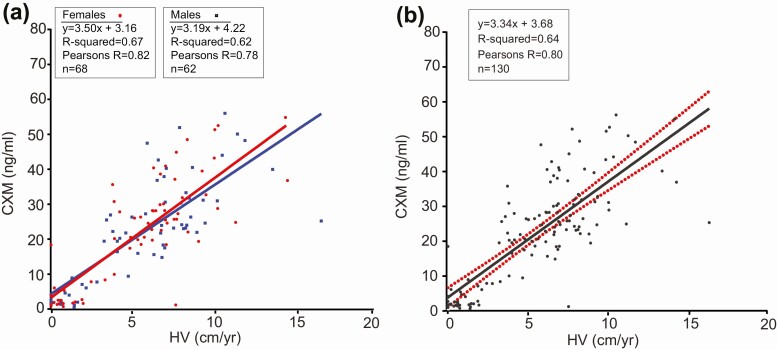
Collagen X biomarker (CXM) concentrations vs height velocity (HV). A, CXM concentrations for males and females plotted vs calculated HVs. Best-fit linear regression lines for both sexes were overlaid on the data. B, Male and female data were combined, and the best-fit linear regression lines were plotted with 95% CI shown in red.

Fig. 4 shows the relationship between CXM and HV values in 110 participants. Compared with our original report, the number of data points is substantially greater (118 vs 44) and the CXM-to-velocity ratio is modestly higher. The slope and corresponding correlation of the HV/CXM lines of best fit for girls is 3.5 CXM ng/mL per cm/year HV (*r* =0.82) compared with 3.19 CXM ng/mL per cm/year HV for boys (*r* = 0.78; Fig. 4A). The Fisher *Z* transformation test found the difference in correlation coefficients was not statistically significant (*z* = 0.6, *P* = .54). Because the difference in slopes was trivial, the results were combined in Fig. 4B.

**Figure 5.**
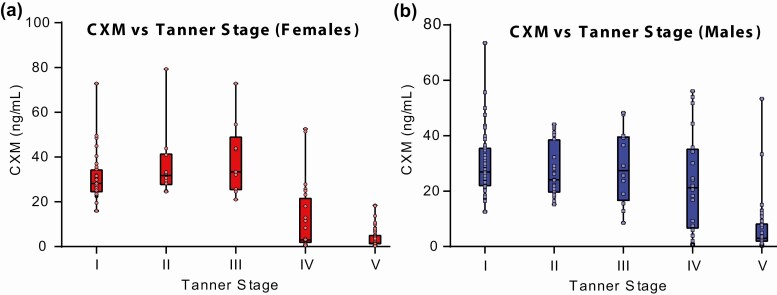
Relationship of collagen X biomarker (CXM) to Tanner stage. CXM levels measured in 199 individuals are displayed according to sex and Tanner stage. For girls, the Kruskal-Wallis overall test was applied (*P* < .001). Dunn test follow-up: peak stage III differed from all other stages (*P* < .05). For boys, the Kruskal-Wallis overall test was applied (*P* < .001). Dunn test follow-up: no statistical difference between stages I to IV and all were higher than stage V (*P* < .001).

Tanner staging was performed on 199 individuals who were at least age 6 years at Nemours and previously described (8). Of the 199, 76 had data from 2 visits, and to maximize sample size the assumption of independence was relaxed and both observations included, yielding a sample of 275 observations (Table 2). The relationship between CXM levels and Tanner stage is shown in Fig. 5. Both for boys and girls, there was an overall difference in CXM by Tanner stage (*P* < .001). For girls, the levels peaked at breast Tanner stage III and are statistically different from girls at all other Tanner stages (*P* < .05 for all Dunn post hoc pairwise comparisons). For the boys, levels were not statistically different between Tanner stages I through IV and all are higher than for boys at Tanner stage V (*P* < .001).

**Table 2.** Distribution of female (F) and male (M) participants by Tanner stage

Tanner	F	M
I	35	47
II	10	23
III	9	12
IV	24	20
V	47	48

**Figure 6.**
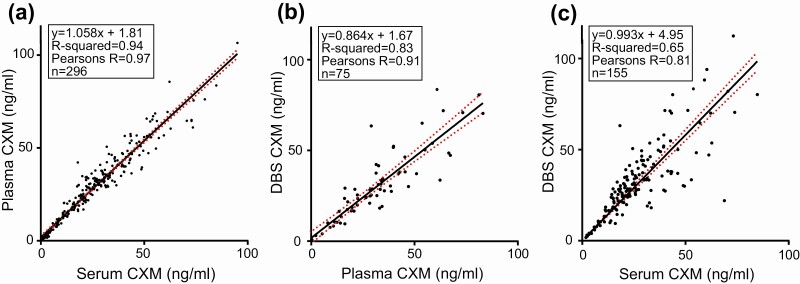
Relationship between serum, plasma, and dried blood spot collagen X biomarker (CXM) concentrations. CXM concentrations of matched samples are compared. The best-fit linear regression line is shown for each comparison in black, with 95% CI shown in red for all comparisons.

New samples included paired serum and plasma, allowing our original comparison of plasma vs serum concentrations of CXM to be expanded and the relationship between the biomarker and the blood component used for assay to be better defined. Fig. 6A shows the similarity of measurements for plasma and serum samples drawn at the same time, indicating that for practical purposes plasma and serum can be used interchangeably for measuring CXM. The majority of samples assayed in this study contained both serum and plasma drawn at the same time for comparison of which the serum value was used to generate normal data in all of the figures. However, only plasma was available for 23 participants and therefore the plasma value was used for analysis. Strong correlations similar to those previously described were observed when retested DBS results were plotted against the combined new and retested plasma and serum biomarker results; therefore, serum and plasma sample data were considered equivalent for this analysis (Fig. 6B and 6C). It is important to note that although the rNC1-spiked controls do not contain serum or plasma, they exhibit coefficient of variation percentages similar to diluted serum and plasma controls, providing evidence that nonspecific binding of reagents or serum effects are minimal in this assay.

## Discussion

We recently identified CXM as a potential biomarker for length/HV from cross-sectional and longitudinal analyses of a relatively small number of healthy children, 83 and 14, respectively (1). Since our original publication, we have optimized the CXM assay, retested our original samples, and added many additional participants, bringing the number of children analyzed to 302 for cross-sectional studies and 116 for longitudinal studies. Fourteen children in the longitudinal study had 2 separate HVs calculated from 3 clinic visits and were therefore used twice to generate 130 data points for Fig. 4. Our new results confirm and strengthen our original findings and support the conclusion that CXM is a real-time biomarker for HV.

Our expanded data set allows for a more complete definition of CXM as a HV marker and its relationship to conventional HV norms derived from stadiometer measurements. For example, conversion of our cross-sectional data to age-specific LMS percentile curves confirms the remarkably similar pattern of CXM plots to conventional HV norms reported by Kelly et al (7). The greater range of CXM values compared with conventional norms presumably reflects the smaller number of data points for CXM so that outliers have a greater impact on curves. This effect is illustrated by the 3 girls with very high CXM values in the 9- to 12-year age range, which shifts the curves upward in Fig. 4.

Analysis of our expanded longitudinal growth velocity data found a strong correlation of CXM with conventional HV, Pearson *r* = 0.80 (*P* < .001) both for girls and boys. Considering that HV is the integrated product of hundreds of genetic and environmental factors, we believe identifying a marker whose level in girls and boys is 69%/59% explained by HV is remarkable. No previously reported biomarker has shown this level of correlation with HV in normally growing children (8, 14).

The reference ranges generated from LMS analysis and plotted in Figs. 2 and 3 are based on a much smaller number of children than are the established norms reported by Kelly and colleagues (7). We view them as low-resolution representations of CXM variation for normally growing healthy children that are expected to evolve as more studies are conducted and definitive norms are established. We consider them an essential step in the development of CXM as a clinical tool to assess HV.

We predict the greatest potential clinical use of CXM will be to monitor changes in HV over time. In contrast to conventional HV determination by stadiometer, which requires taking measurements a minimum of 6 months apart, CXM describes HV in real time. This property makes it potentially ideal for monitoring disease progression or response to growth-stimulating therapies in individual and well-defined cohorts of children in treatment studies. A case in point is the clinical trial recently reported by Savarirayan et al in which children with achondroplasia were treated with daily injections of C-type natriuretic peptide (15). The reported CXM concentrations measured with our assay mirrored dose-dependent increases in conventionally determined HV, the established standard typically required by regulatory agencies, such as the US Food and Drug Administration.

In contrast to the potential utility of CXM in longitudinal studies, the value of single CXM measurements may be limited by the modest variability in the CXM/HV correlation. This variability likely reflects the relatively small number of individuals analyzed to date compared with the large number of children whose growth data were analyzed to establish conventional HV norms (7). Another possible factor is the time of sampling, as we previously described a diurnal variation for CXM with values higher in the morning than in the afternoon (1). Although most of the Nemours samples, which represent most of the samples, were collected in the morning, many of the SHC and OHSU samples were obtained in the afternoon. So diurnal variation may have contributed to the observed variability. We predict that CXM/HV correlation will improve as more samples are analyzed and sampling protocols become more rigorous. If so, one-time CXM measurements may have potential as a real-time screening tool for children whose growth velocity is outside the normal range. Currently, we propose that CXM data reported here be used as working reference ranges for CXM to assess growth in the pediatric population.

There are several technical issues of this study that deserve comment. The first involves how growth velocity is determined and defined. CXM values reflect instantaneous HV at that time of measurement. In contrast, conventional HV values reflect average growth rate during the time period between measurements. The 2 methods are difficult to compare. A useful analogy is measuring blood glucose and glycated hemoglobin in diabetic patients. If one wanted to equate CXM and conventional HV measurements and timing, CXM could be assessed daily for the duration of the measurement period and a value calculated that best correlates with HV from the area under the curve. But this approach is not practical, so we make the assumptions that CXM changes little over a given time span and that a single determination is proportional to the area under the curve, and we use it as a proxy. An exception to this way of thinking may arise during the prepubertal and pubertal years, when sudden changes in HV may occur leading to discrepancy between the 2 methods for tracking HV. Given this possibility, we recommend CXM testing at 2- to 3-month intervals during this period if detecting and/or monitoring the pubertal growth spurt is clinically relevant. Similarly, we suggest that CXM be measured at shorter intervals if it is being used to monitor responses to growth-stimulating therapy. Overall, we stress that CXM measurement and conventional HV determination should be viewed as complementary means to assess HV.

CXM values varied by Tanner stage both for girls and boys in whom staging had been performed (see Fig. 5). The girls peaked at breast Tanner stage III as expected because this coincides with the timing of the pubertal growth spurt. However, we did not find this expected association with boys at Tanner stage IV. We attribute this result to skewing of the data by a small group of Tanner stage IV boys with low CXM levels. We anticipate that boys’ CXM values will end up peaking at genitalia Tanner stage IV as more studies are conducted along these lines.

In our previous publication, the relationship between plasma and serum CXM concentrations suggested that plasma had slightly higher levels potentially due to lost CXM in the removed clot from the blood (1). Testing an additional 217 matched plasma and serum samples to the 115 matched samples from our previous study allowed us to more accurately define the relationship between serum and plasma blood components (see Fig. 6A). Our new data suggest that plasma and serum have equivalent CXM values and therefore either serum or plasma can be used for CXM concentration determinations. Despite this result, we still suggest that if a study is to be performed it use only serum or plasma for the sake of consistency.

When comparing reassayed DBS samples to the reassayed plasma and serum from our previous study, the relationship between each DBS result and its matched plasma or serum counterpart result matched very closely, with similar variance (1). Notably, the values of these rerun samples did not differ significantly from the data generated for our previous publication after being stored at –20°C for more than 2 years since they were previously assayed and more than 5 years since the original sample collection. Similarly, the reassayed values for CXM in plasma and serum compared closely with those obtained for our original publication after being stored for 4 years. Samples assayed from Nemours were collected 8 to 12 years prior and stored at –80°C since collection. The CXM concentration of these samples generated comparable results to those in the same age ranges of our previous study, suggesting that the CXM biomarker is stable for at least this amount of time stored at –20°C and below. Significant amounts of serum and plasma from both of these studies are currently stored at –20°C, so it will be possible to reassay these samples in the future to confirm the stability of this biomarker in samples frozen for prolonged periods. The fact that this biomarker is stable once serum/plasma or DBS is stored at –20°C and below for multiple years may mean that archived samples collected and stored below –20°C for other studies completed long ago may be assayed for the CXM biomarker.

The correlation of CXM measured in DBS to CXM measured in blood, especially serum, is not as strong as it is for CXM measured in serum vs plasma. This is not surprising given the additional steps involved in obtaining and processing DBS samples compared to analyzing serum or plasma samples. Importantly, we have adopted more stringent sampling and processing protocols to minimize DBS variability, which we expect to improve the correlation of DBS to blood CXM measurements going forward.

In summary, we have expanded our observations regarding CXM and HV. We define CXM concentration percentile curves using LMS analysis for normally growing, healthy children from birth to age 20 years. Compared with our initial publication, the substantial increase in sample number allows for more accurate definition and interpretation of CXM levels and percentiles for individuals of a given age and sex. These data provide working reference ranges that can be used to assess individuals with normal and potentially abnormal skeletal growth. We expect CXM to one day become a valuable tool for estimating growth velocity in the clinical setting.

## Data Availability

Some or all data generated or analyzed during this study are included in this published article or in the data repository listed in “References.”

## Abbreviations

CXM: collagen X biomarker
DBS: dried blood spot
ELISA: enzyme-linked immunosorbent assay
HV: height velocity
Nemours: Nemours Children’s Specialty Care
OHSU: Oregon Health & Science University
QC: Quality Control
SHC: Shriners Hospitals for Children

## Additional Information

***Disclosure Summary:*** R.F.C. and W.A.H. are inventors on a patent application, “Type X collagen assay and methods of use thereof,” submitted by Shriners Hospitals for Children. R.F.C. and W.A.H. have consulted for and/or received speaker honoraria from BioMarin and TherAchon (now owned by Pfizer). W.A.H. has also consulted for Ascendis, QED, Relay Therapeutics, Fortress Biotech, OPKO, and Medicell. The other authors have nothing to declare.
